# Metagenome Insights into Armenian Acid Mine Drainage: A Novel Thermoacidophilic Iron-Oxidizing Bacterium with Perspectives for Copper Bioleaching

**DOI:** 10.3390/microorganisms14010146

**Published:** 2026-01-09

**Authors:** Anna Khachatryan, Arevik Vardanyan, Ruiyong Zhang, Yimeng Zhang, Xin Shi, Sabine Willscher, Nhung H. A. Nguyen, Narine Vardanyan

**Affiliations:** 1 Department of Microbiology, SPC “Armbiotechnology” of the National Academy of Sciences of Armenia, 14 Gyurjyan Str., Yerevan 0056, Armenia; anna.khachatryan@asnet.am (A.K.); nvard@sci.am (N.V.); 2 State Key Laboratory of Advanced Marine Materials, Key Laboratory of Marine Environmental Corrosion and Biofouling, Institute of Oceanology, Chinese Academy of Sciences, Qingdao 266071, China; ruiyong.zhang@qdio.ac.cn (R.Z.); zhangyimeng21314@163.com (Y.Z.); shixin@qdio.ac.cn (X.S.); 3 Guangxi Key Laboratory of Marine Environmental Science, Institute of Marine Corrosion Protection, Guangxi Academy of Sciences, Nanning 530007, China; 4 Faculty of Natural Sciences I, Martin Luther University Halle-Wittenberg, 06099 Halle, Germany; sabine.willscher@gmail.com; 5 Institute for Nanomaterials, Advanced Technologies and Innovation, Technical University of Liberec, Bendlova 1409/7, 461 17 Liberec, Czech Republic; nhung.nguyen@tul.cz

**Keywords:** acid mine drainage, metagenomic analysis, microbial biodiversity, metal resistance genes, CAZymes, iron-oxidizing bacteria, copper recovery

## Abstract

The microbial ecology of acid mine drainage (AMD) systems in Armenia, with a long mining history, remains unexplored. This study aimed to characterize the microbial diversity and functional potential of AMD in the Syunik region and to isolate novel microorganisms with biotechnological value. A comprehensive analysis of the microbial communities’ structure of Kavart abandoned, Kapan exploring mines effluent, and Artsvanik tailing was conducted. Metagenomics revealed bacterial-dominated communities, comprising *Pseudomonadota* (previously “*Proteobacteria*”) (68–72%), with site-specific variations in genus abundance. A high abundance and diversity of metal resistance genes (MRGs), particularly for copper and arsenic, were identified. Carbohydrate-active enzyme (CAZy) analysis showed a dominance of GT2 and GT4 genes, suggesting a high potential for extracellular polymeric substances (EPS) production and biofilm formation. A novel strain of iron-oxidizing bacteria Arm-12 was isolated that shares only ~90% similarity with known *Leptospirillum* type species, indicating it may represent a new genus without culturable representatives. The strain exhibits enhanced copper extraction from concentrate. This study provides the first metagenomic insights into Armenian AMD systems and tailing, revealing a unique community rich in metal resistance and biofilm-forming genes. The isolation of a novel highly effective iron-oxidizer Arm-12 highlights the potential of AMD environments as a source of novel taxa with significant applications in biomining and bioremediation processes.

## 1. Introduction

AMD refers to an acidic effluent rich in sulfates and metals. It forms through the microbial oxidation of pyrite and other sulfide minerals, leading to the generation of sulfuric acid and metal-rich solutions. AMD systems are common worldwide, although only a limited number of them have been microbiologically characterized [1,2]. It has recently been demonstrated that some of these AMD formations, such as the Los Rueldos mercury mine in northwestern Spain, appear to be populated by a wide range of prokaryotes, the majority of which are inhabited by a limited set of acidophilic bacteria and archaea, the variety and abundance of which are dependent on geochemical conditions [1,3,4,5,6].

The primary bacterial lineages identified in AMD systems belong to the phyla *Pseudomonadota*, including genera *Acidithiobacillus*, *Acidiphilium*, *Acidocella*, *Ferrovum*, *Acidibacter*, and *Metallibacterium* spp., *Nitrospirae* (*Leptospirillum* spp.), *Firmicutes* (*Sulfobacillus* spp., and *Alicyclobacillus* spp.), and *Acidobacteria* [7,8,9,10,11,12]. In addition, a large number of rare taxa constituting a “rare biosphere” may perform crucial functions in AMD ecosystems have been reported [13]. The *Archaea* include the phylum *Euryarchaeota* (*Ferroplasma* spp.) [14,15,16,17]. These microorganisms are expected to be reservoirs of enzymes adapted to resist acidic conditions [18,19,20], some of which might be of biotechnological relevance.

Detailed reports of processes contributing to AMD generation, including the role of iron- and/or sulfur-oxidizing microbes, are available in the literature [12,21,22,23]. In many AMD sites, oxidation is catalyzed by naturally occurring bacteria, *Acidithiobacillus ferrooxidans* [7,23,24], which accelerate the oxidation reactions for sulfides of most heavy metals. Leptospirilla detected in extremely acidic AMD sites are also an important contributor to the production of AMD [25,26,27,28].

The physicochemical factors influencing microbial community formation in AMD include pH, heavy metals, temperature, dissolved oxygen, total organic carbon (TOC), and solute concentrations. In general, pH is the main factor influencing bacterial diversity. The pH can indirectly impact microorganisms involved in biogeochemical cycles by altering various chemical and physical factors, such as the availability and solubility of metals. The amounts of toxic heavy metals or metalloids influence changes in community structure because heavy metals can bind to microbial proteins, enzymes, and nucleic acids, interfering with their normal functioning and resulting in toxicity [29]. A large number of AMD sites with microbial communities shaped by concentrations of heavy metals/metalloids have been reported [26,27,30]. High concentrations of heavy metals are typically thought to reduce microbial diversity and limit the thriving of microbial communities due to their toxicity [30,31]. However, some microbes demonstrated tolerance towards metals, such as *Acidithiobacillus* spp., *Acidiphilium* spp., and *Leptospirillum* spp. [5,8].

Certain microorganisms can prevent or circumvent the damage effects from heavy metals through the expression of specific metal resistance genes (MRGs). For example, heavy metals are primarily associated with Cu, Zn, Hg, Cr, and Cd in the Pearl River Estuary water [32]. Several studies have reported that heavy metal accumulation may enhance the abundance of resistance genes [33,34].

Bacterial community is also considered a key factor driving the distribution of resistance genes in the environment [35]. The mining activity and accumulation of tailings have led to serious environmental hazards. Despite the global distribution of AMD, its microbial ecology is shaped by local geochemistry [1,2,6]. The microbial ecology of AMD systems in the Caucasus region, particularly in Armenia, with its long history of base and precious metal mining, remains entirely unexplored. This represents a critical knowledge gap, as local geochemistry and mineralogy are key drivers of microbial community structure and function. Consequently, the potential of these unique environments to harbor novel microbial taxa and genetic determinants, such as new MRGs or acid-stable enzymes, is completely unknown. Furthermore, the discovery of novel, robust acidophiles is essential for advancing biomining technologies. Therefore, a comprehensive study of Armenian AMD sites is necessary to understand their specific microbial ecology and to tap into their biotechnological potential. The long-term exposure to heavy metals has affected the biodiversity and composition of microbial communities [36].

In this study, we used molecular analysis to investigate the bacterial community structures in AMD and tailing samples from Syunik Province, Armenia. For the first time, a metagenomic approach was used to investigate the microbial composition of KAM, KEM, and AT to reveal the main taxa and assess the potential of gene sources. Thus, it is critical to illuminate the distribution characteristics of heavy MRGs and their interactions with the bacterial community and environmental factors in AMD and tailing samples.

## 2. Materials and Methods

### 2.1. Collection and Physicochemical Analysis of the Samples

**Figure 1:**
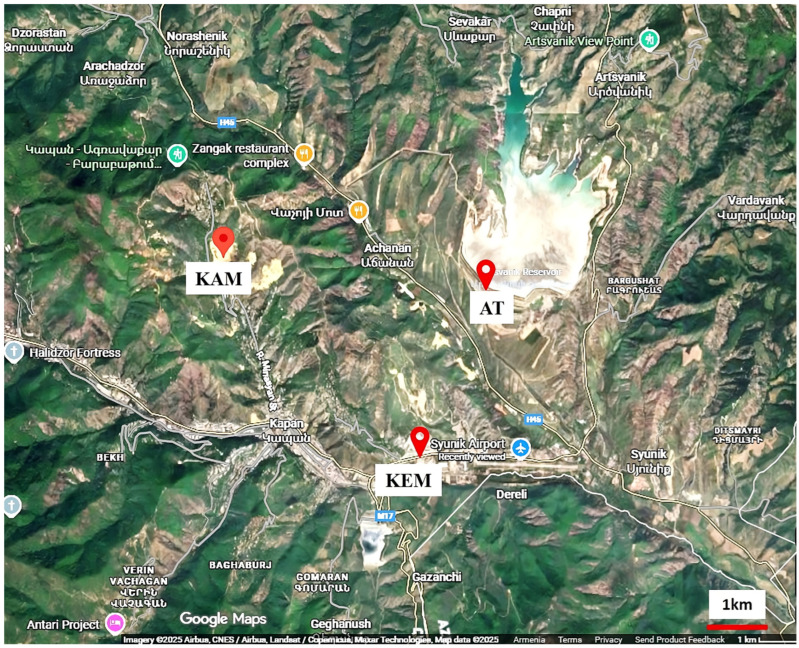
Sites for the collection of AMD and tailing samples for characterization of bacterial communities, structure, and chemical elements.

Kapan mine effluent and Kavart AMD samples, as well as a sample of AT (Syunik region, Armenia), served as subjects for metagenomic analysis. Kapan city is one of the basic industrial cities situated in the southeastern part of Armenia (Figure 1) between the lower streams of Rivers Voghchi and Artsvanik. Kavart is an abandoned mine in the Syunik region. The sampling was carried out in December. Samples of AMD were collected from the mainstream of the Kavart abandoned mine with sediment (KAM) (N39.234800, E46.394133) and from the Kapan exploring mine (mine effluent) (KEM) (N39.203833, E46.429233) (Syunik region, Armenia). The samples were taken into sterile glass bottles. Artsvanik is the largest exploiting tailing located near Kapan city. The sample from AT was collected aseptically from the surface and a 50-cm deep layer of black shale (N39.231433, E46.447067). A total of three samples were collected in July 2023 (summer) and three samples in December 2023 (winter), and were named as KAM, AMD of KEM, and AT. Sampling was performed to account for potential seasonal variation in microbial community composition and geochemical parameters, as AMD systems in continental climates may experience shifts in temperature, hydrology, and metal solubility between warm and cold seasons.

Collecting two time points provided complementary data while maintaining manageable sequencing and analytical effort. Each site was sampled once per season, resulting in a total of six samples, which ensured coverage of the main AMD sources in the region while allowing detailed metagenomic and physicochemical analysis of each sample.

Parameters including the pH, electrical conductivity, redox potential, and total dissolved solids were measured using the multiparameter YSI 556 MPS (Aqua TROLL 500 Multiparameter Sonde, In-Situ Inc., Fort Collins, CO, USA). The samples were analyzed for heavy metal content (Fe, K, Mg, Cu, As, Zn, Al, Co, Ca, Cr, Mn, Se, Ni, Sr, Pb, and Na) using the Agilent 5800 VDV ICP-OES (Inductively Coupled Plasma Optical Emission Spectrometry) (Agilent Technologies, Waldbronn, Germany). Standard solutions were used to make the appropriate calibration curves. Each sample was analyzed in triplicate, and the average absorbance was used to determine the heavy metal concentrations. All samples were kept at 4 ± 0.1 °C in the refrigerator for subsequent molecular biological studies.

### 2.2. DNA Extraction from AMD and Tailing Samples for Metagenomics and Statistical Analysis

The AMD samples of KAM, KEM, and tailing sample AT (each 2 L) were filtered for the metagenome sequencing through a 0.22-μm polycarbonate membrane by using a vacuum filtration system (Millipore Corporation, Billerica, MA, USA). The filtration was performed within 4~8 h, and the filter membranes were quick-frozen in liquid nitrogen and then stored at −80 °C until DNA extraction. DNA extraction for metagenomic studies was performed according to the Fast DNA TMSpin Kit for Soil protocol (MP Bio-Medicals, Santa Ana, CA, USA) according to the manufacturer‘s instructions. The integrity and purity of the DNA were evaluated using 1% agarose gel electrophoresis, while DNA concentration was measured using a Qubit 4.0 fluorometer and a NanoDrop One spectrophotometer (Thermo Fisher Scientific, Waltham, MA, USA). High-quality DNA samples were processed using the ALFA-SEQ DNA Library Prep Kit, following these steps: DNA fragmentation, end repair and 3′ adenylation, adapter ligation, fragment selection, purification, and PCR amplification. The library size distribution was assessed with the Qsep400 High-Throughput Nucleic Acid-Protein Analysis System, and concentrations were measured with Qubit 4.0. Sequencing was performed on an Illumina or MGI platform using paired-end 150 bp (PE150) reads. Raw sequencing reads were processed using Fastp V0.14.1 software to remove low-quality bases and adapters, generating clean reads for downstream analysis. MEGAHIT (v1.2.9) was used for assembly. Scaffolds were broken at N-connection sites to generate scaftigs. Unused reads were pooled and subjected to co-assembly using MEGAHIT, with scaftigs > 500 bp retained for further analysis. Open reading frames were predicted using Prodigal (v2.6.3). The resulting non-redundant gene catalog was clustered at 95% identity using MMseqs2 V16.747c6. Gene abundance was calculated by mapping clean reads to the gene catalog with BBMap. Taxonomic classification was performed using Diamond against the NCBI NR database. The Lowest Common Ancestor algorithm in MEGAN was applied for species annotation. Taxonomic abundance was computed at different levels (Kingdom to Species). For functional analyses, the unique genes were blasted against functional databases, including the CAZy database (Version 2018-012).

### 2.3. Isolation of Arm-12

Samples of AMD of KEM, KAM, and AT were used to establish an enrichment culture of iron-oxidizing bacteria. For this purpose, MAC medium [37] with FeSO_4_ × 7H_2_O as a source of energy at pH 1.85 was inoculated with an AMD sample and incubated at 37 °C and 45 °C for 5–7 days. After enrichment, the target strain was isolated using the serial dilution technique. The enrichment was serially diluted from 10^−1^ to 10^−8^, and all dilutions were subsequently used to inoculate MAC medium at 45 °C. Pure culture Arm-12 was isolated from enrichment culture diluted to 10^−5^ using Manning solid medium with FeSO_4_ × 7H_2_O as a source of energy [38].

### 2.4. Identification of Arm-12

The isolated strain Arm-12 was identified based on the nucleotide sequence analysis of the 16S rRNA gene. The pure culture of Arm-12 in the logarithmic growth phase (106–108) was centrifuged (SIGMA 1-14k, Osterode am Harz, Germany) to collect the bacterial biomass, which was subsequently frozen for DNA purification and taxonomic classification of the strain. DNA extraction was performed following the TIANamp Genomic DNA Kit-DP304 protocol. This DNA kit utilizes silica membrane technology and includes a specialized buffer system for extracting DNA from various sample types. DNA purity and concentration were assessed using a Thermo Scientific™ NanoDrop™ One Microvolume ND-1000 UV-Vis spectrophotometer (NanoDrop Technologies, Wilmington, DE, USA). PCR amplification was performed following the TransStart^®^ FastPfu DNA Polymerase Protocol. The purified PCR products were sequenced using two universal primers: 27F (5′ AGAGTTTGATCCTGGCTCAG) and 1492R (5′ GGTTACCTTGTTACGACTT). The total volume of PCR products was measured using a Thermo Scientific™, NanoDrop™ One Microvolume ND-1000UV-Vis spectrophotometer. The sample was forwarded to Sangon Biotech Co., Ltd. (Shanghai, China) for 16S rRNA gene sequencing.

### 2.5. Phylogenetic Analysis of Arm-12

The initial analysis of the nucleotide sequence similarity of the 16S rRNA gene of the studied strain was conducted using the online NCBI BLAST tool (https://www.ncbi.nlm.nih.gov/blast). To identify phylogenetically related species of the studied bacterium, the nucleotide sequence of the 16S rRNA gene was submitted to the NCBI (National Center for Biotechnology Information) BLAST (https://www.ncbi.nlm.nih.gov/blast) online search system and compared with corresponding sequences in the GenBank database. The obtained 16S rRNA gene sequences of Arm-12 were compared with other 16S rRNA gene sequences available in the EZBioCloud database (https://www.ezbiocloud.net/), and the phylogenetic tree was constructed with MEGA 12 software [39].

### 2.6. Morphology and SEM Studies

Gram-staining of isolated bacteria was performed by the Hucker method [40] and was observed with a Leica DM500 trinocular (×1000) microscope (Leica Microsystems, Wetzlar, Germany). For Scanning Electron Microscope studies (SEM), the pure culture Arm-12 was grown in MAC medium containing 20 g/L of FeSO_4_ × 7H_2_O (analytical grade, VWR Chemicals, Radnor, PA, USA, GPR RECTAPUR^®^) as an energy source. Bacterial biomass was harvested onto a membrane with a pore size of 0.2 μm. The collected biomass was successively dehydrated with ethanol series 30%, 50%, 70%, 80%, 90%, and 96% at 25 °C (2 × 10 min) and stored overnight at 4 °C (1 × 10min) in 30%, 50%, and 100% acetone. The biomass was then dried using critical-point drying and coated with gold. SEM images of the strain were obtained using a Zeiss Sigma 300V P FEG (ZEISS, Oberkochen, Germany) operating at 5 kV.

### 2.7. Optimal Conditions for Growth

The influence of temperature and pH on the growth of the isolated strain Arm-12 was examined in 250 mL Erlenmeyer flasks containing 50 mL of sterile MAC medium with Fe^2+^ as a source of energy on the rotary shaker at 170 rpm (Biossan, Riga, Latvia).

### 2.8. Influence of Metal Ions

The influence of metal ions (Cu^2+^, Mo^2+^, Cr^2+^, Co^2+^, Ni^2+^, and Zn^2+^) on the oxidation of Fe^2+^ by Arm-12 was studied in MAC medium in the different concentration ranges from 5 to 100 mM. The following salfates were used: CuSO_4_ × 5H_2_O (Cu^2+^), Na_2_MoO_4_ × 2H_2_O (Mo^2+^), CrSO_4_ × 5H_2_O (Cr^2+^), CoSO_4_ × 7H_2_O (Co^2+^), NiSO_4_ × 6H_2_O (Ni^2+^), and ZnSO_4_ × 7H_2_O (Zn^2+^). Fe^2+^ and Fe^3+^ ions were determined by the titration method using EDTA [41].

### 2.9. Bioleaching of Copper Concentrate

Copper concentrate in particle size 45 μm produced in Armenia was subjected to research. Bioleaching of copper concentrate was performed using Arm-12 strain. Bioleaching experiments were carried out in 250 mL Erlenmeyer flasks containing 100 mL of MAC medium without iron ions at 45 °C, 150 rpm, 2.5 pulp density (PD). The amount of inoculum for the used cultures was 10%. All experiments were carried out in triplicate. For each bioleaching experiment, chemical controls with the same conditions and without inoculum were included. pH was determined with a pH/mV Meter SevenExcellence (Mettler Toledo, Riga, Latvia) equipped with an Ag/AgCl electrode. The redox potential was measured with a Redox Electrode (Pt/Ag/AgCl) of pH/mV Meter SevenExcellence. Copper and total iron were determined by ICP-OES (Agilent 5800 VDV). Concentrations of Fe^2+^ and Fe^3+^ ions were determined by the complexometric method with EDTA.

## 3. Results

### 3.1. Physicochemical Characteristics of AMD and Tailing Samples

The chemical composition and pH/Eh values of the AMD and tailing samples are shown in Table 1. As shown in Table 1, the samples of AMD (KAM, KEM) and AT were characterized by low pH values: pH 1.8–2.0 and pH 2.5, respectively. The oxidation-reduction potential in the tailing storage facility showed a positive value, indicating the occurrence of an oxidative condition. The results in mine water discharge with high acidity, elevated sulfate concentrations, and significant levels of heavy metals, including As, Pb, Se, Zn, Sr, Cr, Al, Co, Ca, Cu, Mg, Fe, K, Ni, Mn, and Na. Notably, the concentrations of Ca, Fe, Cu, Na, Al, Mg, Zn, and Ca were particularly higher (Table 1).

These conditions, namely low pH, high redox potential, and elevated concentrations of dissolved heavy metals, expedite the oxidation of mine wastes, leading to increased acidity levels [42,43].

### 3.2. Metagenomics Analysis and Microbial Community Composition

The microbial communities of the leakage from KEM, AMD of KAM, and AT samples were mainly dominated by bacteria and accounted for 79.4%, 71.4%, and 87.0%, respectively. The relative abundance (%) of *Archaea* and *Eukaryota* in sample KAM (1.4 and 2.0) was higher compared to samples KEM (0.04 and 0.2) and AT (0.01 and 0.005) (Table 2).

**Figure 2:**
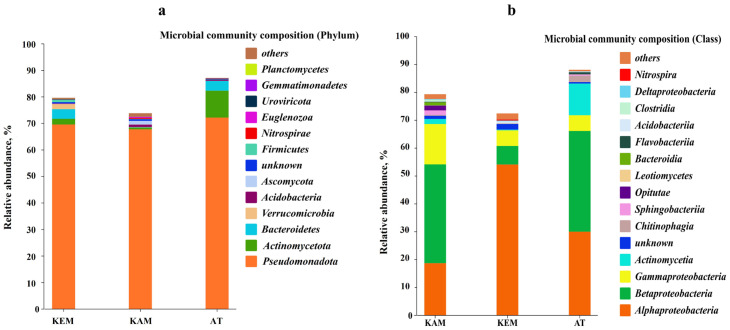
(**a**) The relative abundance of major phyla levels in the microbial communities. (**b**) The relative abundance of major class levels in the microbial communities.

The most abundant phylum that dominated the microbial communities in all three samples was *Pseudomonadota*, accounting for 68–72% of the total taxa, followed by *Actinomycetota* (previously known as “*Actinobacteria*”) and *Bacteroidetes* as the minor phyla level (Figure 2a).

This is consistent with other reports that *Pseudomonadota* were dominant in AMD ecosystems [26,27]. The relative abundance of *Actinomycetota* was higher in sample AT compared to samples KAM and KEM. The most abundant class in KEM and AT was *Betaproteobacteria* (35% in KEM and 36% in AT), while *Alphaproteobacteria* was the most abundant class in the KAM sample with a relative abundance of 54% (Figure 2b). The red color indicates the class *Nitrospira* from which a new genus, Arm-12, was isolated.

**Figure 3:**
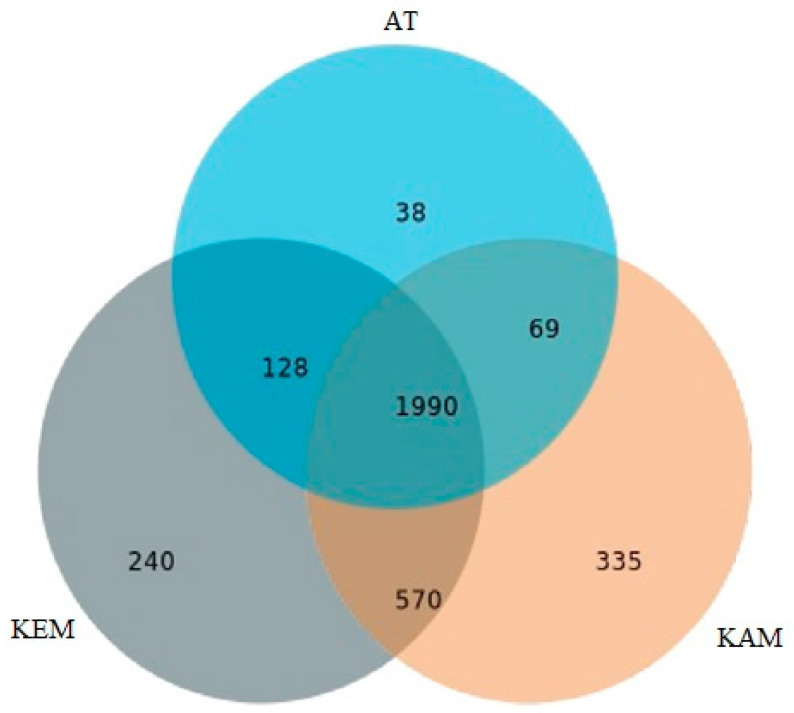
Venn diagram visualizes common genera between the samples (KEM, KAM, and AT).

A high abundance of bacterial species (1990) is shared between the three samples. Following the sharing genera between KEM and KAM with 570, between KEM and AT with 128, and the least is between KAM and AT with only 69 (Figure 3).

The number of genera correlates with the environmental conditions of the sample locations in the order: KAM (335) > KEM (240) and AT (38) (Figure 3).

**Figure 4:**
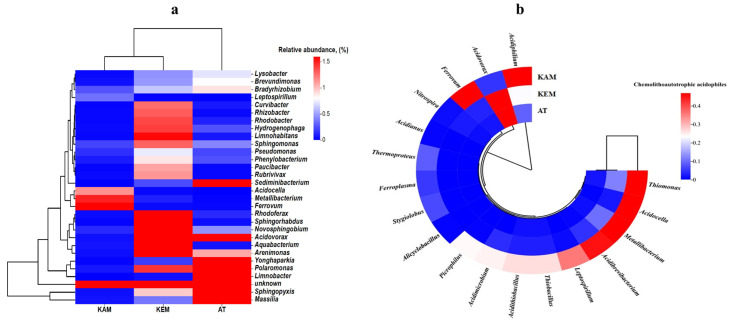
(**a**) A heatmap of the microbial genera in samples KAM, KEM, and AT created on unique gene assignments to the NCBI-NR database. (**b**) The most abundant chemolithoautotrophic acidophiles in samples KAM, KEM, and AT, based on unique gene assignments to the NCBI NR database.

The most abundant genera in all three samples are presented in the heatmap (Figure 4a).

Sample KEM has the highest number of predominant genera (19), followed by AT (9) and KAM (4) (Figure 4a). However, the predominant genera in samples KAM, KEM, and AT were generally distinct. For instance, the unknown genera were the most abundant in KEM, followed by the *Aquabacterium* (4%) and *Arenimonas* (4%). In samples KEM and AT, the unknown genera were 6.2% and 3.2%, respectively. In sample AT, the predominant genus was *Sphingopyxis* with a relative abundance of 22%, followed by *Massilia* (14%) and *Limnobacter* (8%) (Figure 4a). In the KAM sample, the most abundant genera were *Metallibacterium* (1.5%) and *Ferrovum* (2.7%). *Acidocella* accounted for 1.2% of the total microbial taxa (Figure 4a).

As the most common chemolithoautotrophic acidophiles in the KEM sample, *Leptospirillum* species accounted for 0.01% of the total microbial community. In contrast, their relative abundance was 0.4% in the KAM sample and 0.002% in the AT sample. In the KAM sample, the most abundant genus overall was *Acidiphilium*, representing 46% of the total microbial taxa (Figure 4b). Iron-oxidizing acidophiles of *Leptospirillum* and *Acidithiobacillus* genera thriving in extreme environments are the key members of the microbial consortia that are exploited for the industrial recovery of valuable metals such as copper, zinc, and gold [10,30,44,45].

As mentioned above, the most important microbes involved in the bioleaching of minerals are those that are responsible for the production of ferric iron and sulfuric acid required for bioleaching reactions. These include the iron- and/or sulfur-oxidizing chemolithotrophic acidophiles (Figure 4b). Members of the *Ferrovum* genus were detected in abundance in different mining sites and were involved in the oxidation of ferrous iron [46,47,48]. The most abundant genus is *Acidovorax*, accounting for 3.0%, 0.1%, and 2.0% in samples KEM, KAM, and AT, respectively (Figure 4b).

In sample KAM, *Metallibacterium* and *Acidocella* accounted for 1.5% and 1.2% (Figure 4a,b).

Comparative analysis indicated that variations in microbial community composition across KAM, KEM, and AT samples were closely associated with differences in physicochemical conditions. For example, the higher abundance of *Acidiphilium* and *Metallibacterium* in KAM correlated with elevated concentrations of Fe, Cu, and Mn, while the dominance of *Sphingopyxis* and *Massilia* in AT corresponded with relatively higher Zn levels and slightly higher pH (2.5). Similarly, the prevalence of *Aquabacterium* and *Arenimonas* in KEM was associated with moderate Fe and Se levels. Overall, lower pH values (1.8–2.0) supported a higher diversity of acidophiles, whereas increased heavy metal concentrations appeared to shape the abundance of resistant taxa, consistent with observed distributions of metal resistance genes.

*Acidiphilium* and *Acidocella* represent heterotrophic acidophiles that usually share the extreme environment with iron-oxidizing bacteria and form sustainable associations with them. Bacteria showed the ability to reduce ferric ions to ferrous ions when organic substrates were used. Bacteria of the *Acidibrevibacterium* genus are heterotrophic acidophiles isolated from acidic mine drainage [49]. *Metallibacterium* abundant in sample KAM is considered a facultatively anaerobic, acid-tolerant bacterium that could reduce iron [50]. The genus *Acidovorax* is associated with nitrate-dependent Fe (II) oxidation, as four *Acidovorax* species within the class of β-*Proteobacteria* are capable of performing Fe (II)-driven denitrification [51]. These bacteria not only play an important role in biogeochemical iron cycles but also have valuable biotechnological potential applications, such as electronic waste recycling [52,53,54].

The biodiversity of genera could be explained by the chemical profile of samples (Table 1). The majority of bacterial species were found in sample KAM, which contained the highest concentrations of metals compared to samples KEM and AT. Location KAM is characterized by a high abundance of genera *Metallibacterium*, *Acidocella*, *Acidiphilum*, and *Ferrovum*, and contains high concentrations of various heavy metals such as Al, Cu, Cr, and Mn, as well as Ca and Mg (Table 3).

Acidophilic heterotrophic strain GS19h and *Acidocella aminolytica* in the genus *Acidocella*, exhibited high resistance to CdSO_4_, ZnSO_4_, NiSO_4,_ and CuSO_4_ [55]. Other acidophiles resistant to Cd (II) include *Acidiphilum* spp. The above-mentioned species have also been found in other studies, as discussed in the genus-level part [21,46,48,50,56,57]. Genomic sequences of bacteria belonging to the genus *Novosphingobium* found in samples KAM (2%), KEM (0.2%), and AT (0.4%), reveal genes related to heavy-metal resistance [58]. *Sphingomonas* sp. strain DX-T3-03 and *Ralstonia pickettii* strain DX-T3-01 were isolated from the biggest tailing in Asia, located at the Dexing copper mine. The strain DX-T3-01 exhibited high tolerance to Cd^2+^, and the strain DX-T3-03 was highly resistant to Zn^2+^ [59].

### 3.3. Metal Resistance Genes (MRGs)

**Figure 5:**
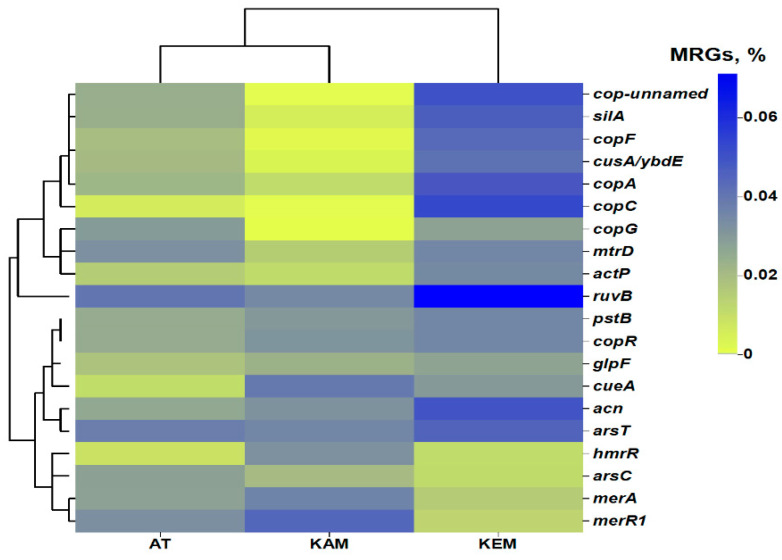
Heatmap of MRGs assigned with NCBI database in KAM, KEM, and AT samples. The value of the color scale represents the relative abundance of functional genes (%).

Metagenomics analysis allowed the description of the most abundant metal MRGs in AMD and tailings samples. The relative abundance of the most identified gene types in each sample was calculated based on the coverage of reads normalized by the total number of reads in metagenomes (±0.2% mean ± standard error of the mean). Thus, the most abundant MRGs were Cu resistance genes (cop-unnamed, *copA*, *copF*, *copR*, *copC copG*, and *ruvB*), the Fe resistance gene (*acn*), As resistance genes (*arsC*, *arsT*, *pstB*), Ag resistance genes (*silA*), and Hg (*merA* and *merR1*). All the detected MRGs subtypes (20 MRGs subtypes) were shared by all KAM, KEM, and AT samples (Figure 5). Cu resistance genes with a high relative abundance of MRGs were discovered, ranging 0.3, 0.1 and 0.2%, followed by MRGs with As resistance (0.1%), Fe resistance (*acn*) (0.05, 0.03 and 0.04%) and Hg (*merA* and *merR1*) (0.012%, 0.044% and 0.0328%) resistance genes in the all metagenomics samples. The mercury reductase enzyme, encoded by *merA* and *merR1* genes, is responsible for the resistance of mercury in microbes.

It has been reported that the *cop* system can be involved in the transport of copper [60] and the removal and detoxification of excess copper from the cell via forming the major copper homeostasis system with other proteins [61]. Besides *actP*, various types of copper resistance genes were also found in the metagenome of samples, such as *ruvB*, cop-unnamed, *copA*, *copF*, *copR*, *copC*, *copG*, and all these genes were involved in the transport of copper. The *actP* encodes copper-transporting P-type ATPase, which is responsible for the efflux pump of copper and multicopper oxidase [62]. In addition, the *ruvB* (0.08%, 0.0344%, and 0.0406%) genes had the highest relative abundance in the sample of KEM, KAM, and AT, respectively (Figure 5). Moreover, *ruvB* is an ATP-dependent DNA helicase that is involved in repairing DNA damage caused by chromate or derivatives [63] and can confer resistance to chromate, tellurite, and selenite. Similar research on active nonferrous metals tailings also revealed that the *genus Thiobacillus* was the main contributor to MRG distributions [64]. The *arsC* and *arsT* genes, which code for an arsenate reductase, are essential for arsenate resistance and transform arsenate into arsenite, which is extruded from the samples. In addition, MRGs (*hmrR*, *glpF*, and *pstB*) related to the resistance of other metals were also observed. Our results show that Cu and As may be the main metals that induce heavy metal resistance genes. These results further support the idea that metals have formative influences on the bacterial community composition as well as metal resistance genes.

### 3.4. CAZy Analysis and Gene Diversity

**Figure 6:**
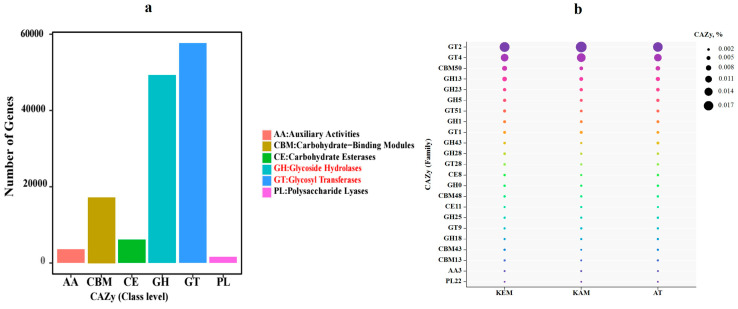
(**a**) Statistical plot of annotated results of CAZy enzymes: AAs, CBMs, CEs, GHs, and PLs finding modules, (**b**) bubble plot of functional genes encoding CAZy enzymes relative (%) in KAM, KEM, and AT. The X-axis represents samples, and the Y-axis represents the most CAZy encoded family by identified functional genes.

The metagenomics data revealed the most abundant CAZy in AMD (KAM, KEM) and tailing (AT) samples. According to the metagenomics results of total contigs against the CAZy database, combined with the hierarchy structure of the CAZy database. The data show the most abundant CAZy enzyme class (AA: Auxiliary Activities, CBM: Carbohydrate-Binding Modules, GT: Glycosyl Transferases) encoding genes identified in three metagenomics samples. Comparing the CAZy database revealed the GTs family had the most genes (57.656), followed by the family of GHs genes (49.224), CBMs family genes (17.207), CEs family genes (6.061), AAs family genes (3.572), and PLs family genes (1.532) (Figure 6a).

The most abundant enzyme families predicted in this genome were GT2, GT4, CBM50, GH13, GH23, GH5, GH38, GT51 and GH1 from the CAZymes database (Figure 6b). The most abundant enzymes in KEM, KAM, and AT samples are the GT2 and GT4 families, which account for 1.8, 2.0, 1.7% and 1.3, 1.5, and 1.3%, respectively. CBM50 family accounts for 0.72, 0.45, and AT 0.62% (±0.2% mean ± standard error of the mean), respectively (Figure 6b).

As mentioned above, the most abundant class is GT, which is involved in the biosynthesis of oligosaccharides, disaccharides, and polysaccharides by catalyzing the transfer of glycosylates from activated donor molecules to specific receptors to form glycosidic bonds. The most widely distributed GT2 family proteins have the activities of chitin, cellulose, hyaluronic acid, and glucan synthetase. Among the 42 CBM families found, the functions detected were cellulose, chitin, galactan, galactose, glycogen, pectin, starch, and xylan binding. The main role of EPS is the mediation of the initial attachment of cells to solid substrates and protection against harmful environmental factors. Acidophilic microorganisms have the capability to multiply, attach to solid and liquid surfaces, and produce a matrix of EPS, forming a biofilm that includes lipids, glycolipids, lipopolysaccharides, proteins, carbohydrates, uronic acids, and nucleic acids [10]. It is assumed that during their growth, bacterial cells form biofilm consisting of EPS, which significantly increases the resistance of bacteria to heavy metals. GT4, GT2, glycoside hydrolase responsible for the hydrolysis and/or regrouping of glycosidic bonds.

The GH family can hydrolyze the glycosidic bonds between two or more carbohydrates or between carbohydrate and non-carbohydrate components, and is essential in the red algae polysaccharide hydrolysis process [65]. In addition to GTs, GHs such as GH10, 13, and 23 that can degrade polysaccharides with alpha-1,4-glycosidic bonds into available carbon sources for bacteria in biofilms have also been identified. The identification of a high abundance of genes encoding GT2 and GT4 indicated their potential roles in constructing EPS by producing various polysaccharides to form biofilms.

### 3.5. Phylogenetic Analysis of 16S rRNA and Identification of a New Strain

The newly isolated iron-oxidizing Arm-12 was obtained from the KAM sample. The length of sequenced fragments of the gene encoding 16S rRNA is 1433 bp. The nucleotide sequence of the strain Arm-12 was phylogenetically compared with those of the 14 species.

**Figure 7:**
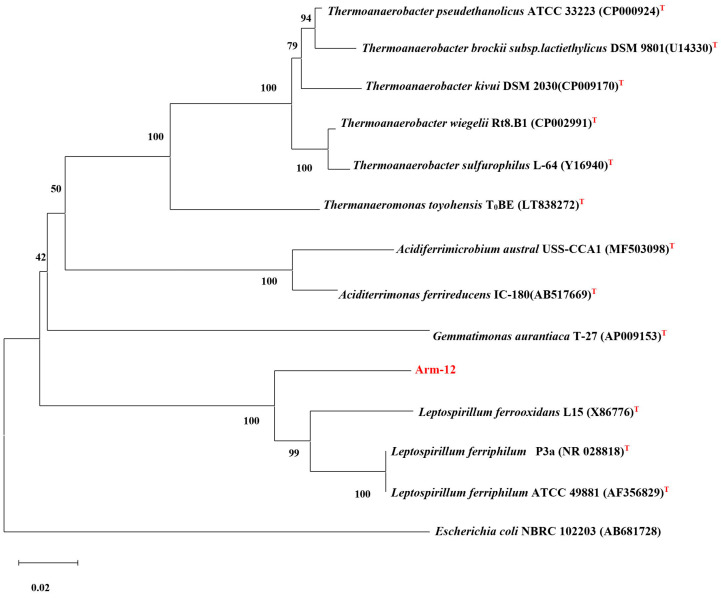
Phylogenetic position of the strain of Arm-12. The superscript “T” indicates a type strain.

The screening of the NCBI and EZBioCloud database (https://www.ezbiocloud.net/) was performed using BLAST (Bioproject). Based on the homology of the 16S rRNA gene, the phylogenetic development tree was built (Figure 7). The sequences of the strain Arm-12 were phylogenetically compared with those of the *Leptospirillum* species. As shown in the dendrogram, the isolate Arm-12, compared to other strains *Leptospirillum ferriphilum* ATCC 49881 and P3a, exhibited 90.77% and *Leptospirillum ferrooxidans* L15—89.54% similarity, respectively (Figure 7).

The 16S rRNA gene sequence was submitted to GenBank, and the accession number PP389931 was assigned. Thus, a novel iron-oxidizing bacterium, Arm-12, which shares only 90% similarity with *Leptospirillum* spp., may represent a new genus without culturable representatives.

New isolate highlighted in red. The evolutionary history was inferred using the neighbor-joining method. The percentage of replicate trees in which the associated taxa clustered together in the bootstrap test (10,000 replicates) is shown next to the branches.

### 3.6. Cell Morphology and Growth Conditions

**Figure 8:**
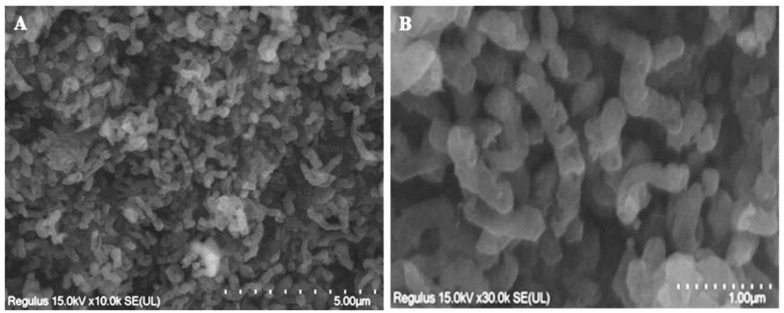
SEM micrograph of the pure culture of an Arm-12. Bar represents 5 μm (**A**), 1 μm (**B**).

As depicted in Figure 8, the Arm-12 strain cells are motile and exhibit a vibrio- or spiral shape, with a diameter of 0.6 μm and a length ranging from 0.8 to 2.6 μm. In younger cultures, the cells are predominantly vibrio-shaped, while in older cultures (after 7 days), they become partially spiral-shaped with two to three turns. The cell wall of the Arm-12 strain was typical for Gram-negative bacteria.

**Figure 9:**
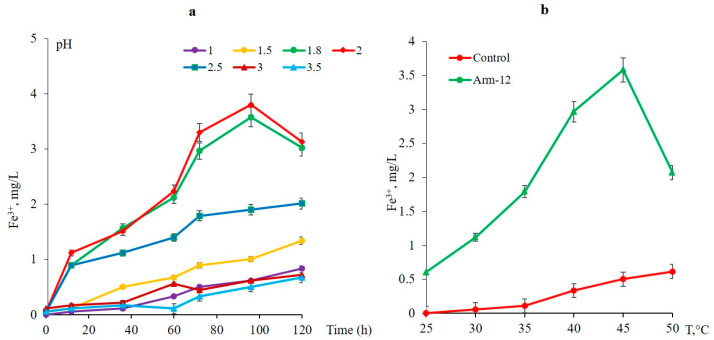
(**a**) Effect of different pH on the growth of the Arm-12 strain (45 °C, 170 rpm). (**b**) Effect of temperature on the growth of the Arm-12-strain (pH-1.9, 170 rpm).

Figure 9a illustrates the effects of pH on the growth of Arm-12. The optimal pH range is 1.8–2.0, where the cell density reaches its peak, and Fe^2+^ oxidation is 3.0–3.5 mg/L. At lower pH levels, bacterial growth and Fe^2+^ are inhibited.

The growth of the isolated Arm-12 was studied in the temperature range of 25–50 °C. The maximum growth and Fe^2+^ oxidation rates of the new isolate were observed at 45 °C (Figure 9b). The obtained results, in general, were comparable with other strains available in the laboratory. However, the Arm-12 exhibits a higher temperature optimum in comparison with previous isolates [10,12].

### 3.7. Influence of Heavy Metal Ions of Leptospirillum sp. Arm-12

The influence of Cu^2+^, Mo^2+^, Cr^2+^, Co^2+^, Zn^2+^, and Ni^2+^ ions on the oxidation of Fe^2+^ by the new genus Arm-12 was studied. The metal ion concentration ranged from 5 to 100 mM. According to data presented in Table 4, the toxicity of the tested ions for isolated *Leptospirillum* sp. Arm-12 was found to be as follows: Mo ˃ Cr ˃ Co ˃ Ni ˃ Zn ˃ Cu. Minimum inhibitory concentrations (MICs) of the tested metals for Arm-12 were determined. MICs were 5 mM for Mo, Cr, Co, Zn, and Ni.

However, in the case of Ni and Zn, the isolated bacteria showed higher iron oxidation activity, 67% and 76%. It is worth mentioning that the MIS for Cu was 10 mM. Thus, isolated bacteria showed high tolerance for Cu^2+^, which gives the strain Arm-12 a definite advantage in bioleaching of copper minerals, including chalcopyrite.

### 3.8. Bioleaching of Copper Concentrate by Leptospirillum sp. Arm-12

**Figure 10:**
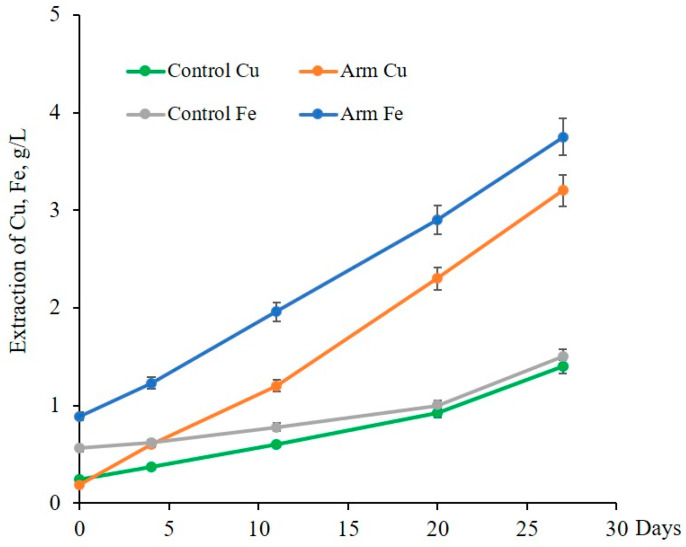
Extraction of copper and iron during bioleaching of copper concentrate by native association of iron-oxidizing bacteria Arm-12 (PD-2.5%, 150 rpm, 45 °C).

To assess the potential of isolated iron-oxidizing bacteria *Leptospirillum* sp. Arm-12 in the biotechnology of metals, it was tested for bioleaching of copper concentrate. It was revealed that, although with a long lag period, isolated bacteria were capable of recovering copper and iron from copper concentrate. Thus, the data presented in Figure 10 show that Arm-12 accelerated the extraction of copper and iron about 2.3–2.5 and 2.5–2.9 times, respectively, compared to the uninoculated control (Figure 10). At 45 °C, copper could be completely dissolved in 27 days. It is concluded that moderate thermophilic *Leptospirillum* sp. Arm-12 isolated from the KAM sample can be successfully used for sustainable copper extraction processes.

## 4. Discussion

Performed molecular biological analysis revealed that microbial communities in AMD and tailings samples (Syunik Region) were dominated by *Pseudomonadota*, with *Actinomycetota* and *Bacteroidetes* as minor contributors. This finding is consistent with literature data on AMD ecosystems in Spain, China, and South America [1,8,26]. However, the genus-level profiles showed unique features. Thus, the dominance of *Sphingopyxis* and *Massilia* in the AT contrasts with the prevalence of *Leptospirillum* and *Acidithiobacillus* typically reported in highly acidic AMD streams [2,5]. Similarly, the high abundance of *Acidiphilium* and *Metallibacterium* in KAM suggests adaptations to elevated Fe, Cu, and Mn concentrations, consistent with studies from polymetallic sites in Canada and China [27,64]. These comparisons indicate that the Armenian AMD communities are shaped strongly by local geochemical conditions, supporting the idea of site-specific ecological structuring.

Our findings demonstrate distinct genus-level variations across sites, likely influenced by differences in heavy metal concentrations, a pattern that has not been extensively documented in Armenian AMD environments. It is worth mentioning that bacteria discovered as predominant genera in tested AMD and tailing samples were reported to be involved in the transformation of elements in nature, including various metals. Most species, particularly acidophiles, exhibit the ability to both reduce ferric iron and oxidize ferrous iron depending on prevailing environmental conditions.

Representatives of the genus *Metallibacterium* abundant in sample KAM were also isolated from an acidic biofilm (pH 2.5) formed on pyrite surfaces and were considered as facultatively anaerobic, acid-tolerant bacteria. It was reported that *Metallibacterium scheffleri* strain DKE6 (T) could reduce iron but was not able to oxidize iron or thiosulfate [50]. Among iron-oxidizing bacteria playing a key role in the natural attenuation of arsenic in AMDs, members of the *Ferrovum* genus were also detected in abundance across different mining sites and identified in mine effluent and water treatment plants [46,47,48]. *Fv. myxofaciens* is less acidophilic than *Acidithiobacillus* spp. and appears to oxidize ferrous iron only. *Fv. myxofaciens* is widely distributed in acidic, iron-rich streams and rivers as macroscopic streamer growth [66]. It has also been identified as the major iron-oxidizing bacterium colonizing a pilot-scale mine water treatment plant designed to oxidize and precipitate iron from contaminated groundwater [67].

Acidophilic heterotrophs belonging to the *Acidiphilium* and *Acidocella* genera inhabit strongly acidic mineral environments and are frequently found in tight association with iron-oxidizing *Acidithiobacillus* spp. Bacteria reduced ferric ions to ferrous ions when organic substrates were used under both aerobic and anaerobic conditions. In addition, it has been found that heterotrophic acidophiles (*Acidiphilium* and *Acidocella*) can biodegrade organic compounds as well as cell lysis (CLs) products and alleviate the toxic effect of CLs products on autotrophic iron-oxidizing bacteria *A. ferrooxidans. L. ferrooxidans* [68]. Genus *Yonghaparkia* (Phylum *Actinomycetota*) found in the AT sample was isolated from microbial communities in a gold mine (Linglong, China) [69], and can contribute to the biogeochemical cycles of S and Fe. *Sphingopyxis* sp. QXT-31 was reported to reduce arsenate (As (V)) to arsenite (As (III)) and oxidize Mn (II) [70]. *Sphingopyxis* spp. were isolated from diverse niches, including agricultural soil, marine, and freshwater, caves, activated sludge, thermal springs, and metal-contaminated sites. *Sphingopyxis* species have drawn considerable attention for not only their ability to survive under extreme environments but also for their potential to degrade a number of environmental contaminants that impose a serious threat to human health. Genus *Polaromonas* was potentially responsible for V^V^ reduction [71]. Gram-negative bacteria in the genus *Aquabacterium* are involved in iron metabolism and can couple iron oxidation and nitrate reduction in anaerobic environments [72].

A notable feature of the microbial communities across the studied AMD sites is the relatively high abundance of sequences assigned to unclassified or “unknown” genera, particularly in the KEM and AT samples. Rather than representing a methodological limitation alone, this pattern likely reflects the extreme and highly selective nature of AMD ecosystems. The combination of very low pH (1.8–2.5), elevated metal concentrations, and limited organic carbon availability creates strong evolutionary pressure favoring specialized metabolic strategies and niche adaptation.

Metagenomics analysis allowed the description of the most abundant MRGs in AMD and tailings samples. Cu resistance genes with a high relative abundance of MRGs were discovered, followed by MRGs with As resistance, Fe resistance, and Hg resistance genes in all metagenomics samples. Our results show that Cu and As may be the main metal elements that induce heavy metal resistance genes. The high relative abundance of Cu- and As-related resistance genes suggests that these metals are dominant selective pressures in our study sites. This is consistent with metal profiles of the samples and aligns with reports linking heavy metal exposure to enrichment of resistance determinants in AMD and contaminated soils [33,34].

The metagenomics data revealed the abundance of CAZy in AMD (KAM, KEM) and tailing (AT) samples. The data show the most abundant CAZy enzyme class (AAs, CBMs, GTs) encoding genes identified in all tested samples. Identification of a high abundance of genes encoding GT2 and GT4 indicated their potential roles in constructing EPS by producing various polysaccharides to form biofilms. The identification of GT2 and GT4 glycosyl transferases highlights the importance of EPS production, which facilitates both survival under metal stress and colonization of mineral surfaces—a prerequisite for bioleaching activity [10]. We focused our functional analysis on MRGs and CAZy because they represent two key adaptive strategies in AMD systems: (i) detoxification and homeostasis of toxic metals, and (ii) EPS biosynthesis, enabling biofilm formation and protection against stress.

Notably, a novel thermophilic iron-oxidizing *Leptospirillum* sp. Arm-12 (PP389931) was isolated, exhibiting a higher optimal growth temperature (45 °C) compared to previously described strains [12]. The isolate Arm-12, compared to *Leptospirillum ferriphilum* ATCC 49881 and *Leptospirillum ferrooxidans* L15, exhibited 90.77% and 89.54% similarity, respectively. Thus, a novel iron-oxidizing bacterium, Arm-12, which is only 90% similar to *Leptospirillum* spp., may belong to a new genus without culturable representatives. So far, most studies have identified *Leptospirillum* spp. bacteria as key contributors to iron oxidation in AMD environments [2,16,17,73]. Moreover, our studies demonstrated that Arm-12 possesses enhanced adaptability to extreme conditions (low pH, high temperature), suggesting its potential for bioleaching applications in elevated temperature, acidic environments. The isolation of the novel bacterium Arm-12 further emphasizes the untapped microbial diversity and biotechnological potential of these ecosystems.

## 5. Conclusions

This study provides the first molecular insight into the microbial communities of AMD ecosystems in Armenia. We have demonstrated that the unique geochemistry of the Syunik region supports distinct microbial assemblages, rich in MRGs and enzymes for biofilm formation, underscoring their adaptive strategies to extreme metal stress. It was revealed that the microbial communities of the leakage from the KEM, the AMD of KAM, and the AT samples were predominantly composed of bacteria. The three samples have a significant abundance of 1990 bacterial species in common, with additional genera shared by KAM and AT, KAM and KEM, and KAM and AT. The major taxa in samples KAM, KEM, and AT were generally distinct, which might be correlated to the chemical composition of the samples.

The high relative abundance and diversity of Cu- and As-related resistance genes across all samples indicate that these metals are major drivers influencing both the structure of microbial communities and the distribution of MRGs in metal-contaminated sites.

The metagenomics revealed CAZy in AMD and tailing samples. The most abundant enzymes in the samples are GT2 and GT4 families. The dominance of GT2 and GT4 glycosyl transferase genes across all samples highlights their central role in polysaccharide biosynthesis for EPS matrix formation, which supports microbial adhesion, biofilm development, and resistance to environmental stress in metal-contaminated ecosystems.

A significant finding of this work is the isolation and preliminary characterization of the novel bacterium Arm-12 (PP389931). Its low 16S rRNA gene sequence similarity (90% related to *Leptospirillum*) suggests it may represent a new genus without culturable representatives. The physiological characteristics of Arm-12, its thermophily, extreme acidophily, and high copper tolerance, coupled with its efficacy in enhancing copper recovery, position it as an extremely promising candidate for the development of more efficient and robust biomining processes.

The characterization of metal resistance determinants will not only contribute to our understanding of the mechanisms that acidophilic bioleaching and other microorganisms use to adapt to their extreme environments, but also to eventually improve biomining or metal bioremediation processes as well.

This study represents the innovative investigation of microbial communities in AMD and tailing samples from Armenia using the molecular biological approach, revealing unique site-specific microbial diversity influenced by heavy metal concentrations. These results provide new insights into the microbial ecology of AMD environments and contribute valuable data to the study of acidophilic metal-tolerant bacteria to expand the current understanding of AMD microbiomes. This research not only expands our understanding of microbial diversity in extreme environments but also opens new avenues for biotechnological applications as well as the development of new AMD remediation strategies by leveraging the untapped potential of unique geographical sites. The isolation and further understanding of anaerobic acidophiles will allow us to propose a methodology for selectively precipitating toxic metals from AMD, transforming this pollution problem into a potential source of metals.

## Figures and Tables

**Table 1:** Physicochemical characteristics of the sampling area *.

Samplings Sites	First Sampling		
	KEM	KAM	AT
	Summer 2023		Winter 2023
Sampling locations (GPS)	N39.203833, E46.429233	N39.234800, E46.394133	N39.231433, E46.447067
pH/Eh	2.0/630	1.8/580	2.5/430
Temperature (°C)	15	14	8
Chemical elements	Chemical composition (=mg/kg = mg/L)		
Al	0.61 ± 0.037	61.0 ± 4.550	0.93 ± 0.004
Ca	155.0 ± 1.030	259.0 ± 5.194	3.8 ± 0.111
Co	0.02 ± 0.009	0.14 ± 0.001	0.01 ± 0.009
As	0.02 ± 0.009	0.01 ± 0.009	0.01 ± 0.009
Cr	0.01 ± 0.001	0.09 ± 0.009	0.01 ± 0.009
Cu	0.03 ± 0.001	11.6 ± 0.694	0.1 ± 0.009
Fe	0.9 ± 0.005	52.0 ± 1.888	0.6 ± 0.010
K	7.2 ± 0.620	2.8 ± 0.276	1.2 ± 0.045
Mg	27.4 ± 0.280	129.6 ± 1.087	2.9 ± 0.103
Mn	0.27 ± 0.020	5.82 ± 0.727	0.11 ± 0.010
Na	1.4 ± 0.064	1.04 ± 0.025	0.02 ± 0.009
Ni	0.02 ± 0.009	0.11 ± 0.008	0.01 ± 0.009
Pb	0.1 ± 0.024	0.16 ± 0.012	0.06 ± 0.009
Se	0.12 ± 0.005	0.04 ± 0.008	0.05 ± 0.002
Sr	0.62 ± 0.049	0.71 ± 0.034	0.8 ± 0.055
Zn	17.2 ± 1.070	30.1 ± 1.64	39.7 ± 0.579

* Values are presented as mean ± standard deviation of triplicate measurements (n = 3).

**Table 2:** The relative abundance of the microbial kingdoms in samples KAM, KEM, and AT was determined based on unique gene assignments to the NCBI-NR database (%).

Kingdom	KEM	KAM	AT
Unknown	0.2	0.08	0.2
Archaea	0.04	1.4	0.01
Bacteria	79.4	71.4	87.0
Eukaryota	0.2	2.0	0.005

**Table 3:** Summary of the correlation of selected significant species in three sample locations (KAM, KEM, and AT). The metal concentration is ≥0.1 mg/kg (details in Table 1).

KEM (Al, Fe, Mg, Mn, Se, Pd)		AT (Al. Fe, Mg, Mn)		KAM (Al, Cu, Fe, Mg, Mn, Ni, Pb)	
Genus	Species	Genus	Species	Genus	Species
*Arenimonas*	*Arenimonas metallic*	*Limnobacter*	*Limnobacter* sp. MED105	*Acidiphilum*	*Acidiphilium angustum*
*Aquabacterium*	*Aquabacterium pictum*		*Limnobacter* sp. 130		*Acidiphilium iwatense*
*Betaproteobacteria*	*Rhodoferax* sp. AJA081-3		*Limnobacter alexandrii*		*Acidiphilium multivorum*
*Fluviicoccus*	*Fluviicoccus keumensis*	*Massilia*	*Massilia* sp. BSC 265		*Acidiphilium rubrum*
*Novosphingobium*	*Novosphingobium* *ginsenosidimutans*	*Sediminibaterium*	*Sediminibacterium* *goheungense*		*Acidiphilium* sp. 37-64-53
*Optutus*	*Optitus* sp. GAS368	*Polaromonas*	*Polaromonas* sp. AER18D-145		*Acidiphilium* sp. 21-60-14
*Prevotella*	*Prevotella copri*	*Sphingopyxis*	*Sphinogopyxis bauzanensis*	*Metallibacterium*	*Metallibacterium* *scheffleri*
*Sphingomonas*	*Sphingomonas lacunae*			*Ferrovum*	*Ferrovum myxofaciens*
*Sphingopyxis*	*Sphingopyxis* sp. L1A2A				

**Table 4:** The influence of metal ions on Fe^2+^ oxidation by *Leptospirillum* sp. Arm-12.

N	Metal Ions, mM	Fe, Oxidized, 3–5 Days											
		Cu^2+^ (CuSO_4_ × 5H_2_O)		Mo^2+^ (Na_2_MoO_4_ × 2H_2_O)		Cr^2+^ (CrSO_4_ × 5H_2_O)		Co^2+^ (CoSO_4_ × 7H_2_O)		Zn^2+^ (ZnSO_4_ × 7H_2_O)		Ni^2+^ (NiSO_4_ × 6H_2_O)	
		g/L	%	g/L	%	g/L	%	g/L	%	g/L	%	g/L	%
1	Control	4.2	100	4.2	100	4.2	100	4.2	100	4.2	100	4.2	100
2	5	3.9	92.8	1.2	29.0	0.80	19.0	1.6	33.0	2.8	67.0	3.2	76.1
3	10	3.2	76.1	0.8	19.0	0.5	12.0	1.2	25.0	2.1	50.0	2.8	67.0
4	25	2.5	60.0	0.5	12.0	-	-	0.8	16.3	1.6	33.0	2.0	48.0
5	50	1.5	36.0	0.5	12.0	-	-	0.5	12.0	1.0	24.0	1.2	25.0
6	100	0.8	16.3	-	-	-	-	-	-	0.5	12.0	0.8	16.3

## Data Availability

The raw sequencing data have been deposited in the NCBI Short Read Archive (SRA) under BioProject accession number PRJNA1247771, with associated BioSample numbers (BioSample IDs: SAMN47967554; SAMN47967555; SAMN47967556). The 16S rRNA gene sequence of Arm-12 was submitted to GenBank and assigned the accession number PP389931.

## Abbreviations

The following abbreviations are used in this manuscript:

AA	Auxiliary Activities
AMD	Acid Mine Drainage
AT	Artsvanik Tailing
CAZy	Carbohydrate-Active Enzymes
CBM	Carbohydrate-Binding Modules
CE	Carbohydrate Esterases
CLs	Cell Lysis
EDTA	Ethylenediaminetetraacetic acid
EPS	Extracellular Polymeric Substances
GH	Glycoside Hydrolases
GT	Glycosyl Transferases
ICP-OES	Inductively Coupled Plasma Optical Emission Spectrometry
KAM	Kavart Abandoned Mine
KEM	Kapan Exploring Mine
MAC	Mackintosh
MRGs	Metal Resistance Genes
PD	Pulp density
PL	Polysaccharide Transferases
SEM	Scanning Electron Microscope
TOC	Total Organic Carbon
